# Glycoproteins C and D of PRV Strain HB1201 Contribute Individually to the Escape From Bartha-K61 Vaccine-Induced Immunity

**DOI:** 10.3389/fmicb.2020.00323

**Published:** 2020-03-10

**Authors:** Jianle Ren, Haibao Wang, Lei Zhou, Xinna Ge, Xin Guo, Jun Han, Hanchun Yang

**Affiliations:** Key Laboratory of Animal Epidemiology of the Ministry of Agriculture and Rural Affairs, and College of Veterinary Medicine, China Agricultural University, Beijing, China

**Keywords:** PRV variant, gC, gD, Bartha-K61, CRISPR/Cas9, genetic variation, immune escape

## Abstract

The newly emerged pseudorabies virus (PRV) novel variants can escape from the immunity induced by the classical vaccine Bartha-K61. Here we investigated the underlying mechanisms by constructing chimeric mutants between epidemic strain HB1201 and the Bartha-K61 vaccine. Our analyses focused on three viral envelope glycoproteins, namely gB, gC, and gD, as they exhibit remarkable genetic variations and are also involved in induction of protective immunity. The corresponding genes were swapped reciprocally either individually or in combination by using CRISPR/Cas9 technology and homologous recombination. The rescued chimeric viruses exhibited differential sensitivity to neutralizing antibodies *in vitro*, and gC was found to be the major contributor to inefficient neutralization against HB1201 by anti-Bartha-K61 serum. When tested in the 4-week-piglet model, substitution with HB1201 gC enabled Bartha-K61 to induce a protective immunity against HB1201 at a high challenge dose of 10^7^ TCID_50_. Interestingly, despite a relatively lower cross-neutralization ability, the gD exchange also enabled Bartha-K61 to protect piglets from lethal challenge. In both cases, clinical signs and microscopic lesions were eased, and so was the viral tissue load with the exception of brain. A better protection could be achieved when both gC and gD were swapped in terms of reducing viral load in brain and virus-induced microscopic lesions. Thus, our studies not only revealed individual roles of gC and gD variations in the immune escape and also suggested a synergistic effect of both proteins on induction of protective immunity. These findings have important implications in novel vaccine development for PRV control in China.

## Introduction

Pseudorabies virus (PRV) is the etiological agent of pseudorabies (PR) or Aujeszky’s disease that was first described in 1813 (Lee and Wilson, 1979). Phylogenetically, this enveloped double-stranded DNA virus is an alphaherpesvirus that belongs to the genus *Varicellovirus* within the family *Herpesviridae* (Mettenleiter, 2000; Pomeranz et al., 2005; Muller et al., 2011; An et al., 2013). PRV has the ability to infect a variety of animal species (e.g., ruminants, carnivores and rodents, etc.) with often-lethal consequences, but pigs are the only natural host (Freuling et al., 2017;Ai et al., 2018). Infections of pigs by PRV can cause reproductive failure of sows and high mortality of young piglets, often leading to colossal economic losses to the swine industry (Muller et al., 2011). Currently, the disease control is carried out mainly through vaccination using attenuated gene-deletion vaccines along with differential diagnosis to differentiate vaccinated from infected animals (Muller et al., 2011; Freuling et al., 2017). Since the implementation of this strategy in 1980s, PRV has been successfully eradicated from domestic pigs of many countries, including the United States, most of Europe and New Zealand (Pannett et al., 1999; An et al., 2013; Ketusing et al., 2014; Freuling et al., 2017). For the Chinese swine industry, Bartha-K61 vaccine has been widely used since early 1990s (An et al., 2013; Sun et al., 2016); it was imported from Hungary in 1979 that contains a complete deletion of the region coding for glycoprotein E (gE) and US9 and partial deletion within the gI and US2 coding regions (Bartha, 1961; Yuan et al., 1983; Lomniczi et al., 1984). Massive vaccination with the Bartha-K61 vaccine in swine farms has brought PR well under control, except sporadic cases (Tong and Chen, 1999; An et al., 2013; Sun et al., 2016).

Nevertheless in late 2011, a severe form of PR emerged suddenly in Bartha-K61-vaccinated swine farms in North China and then quickly spread to most parts of the mainland (An et al., 2013; Yu et al., 2014). Clinical presentation of the disease was manifested by increased abortion rate of sows and nearly 100% mortality in young pigs. Additionally, it caused substantial death to fattening pigs (An et al., 2013; Yu et al., 2014; Tong et al., 2015; Yang, 2016; Yang et al., 2016). The causative agent is a type II PRV novel variant, and the representative strains are HB1201, HeN1, HLJ-8, TJ, HN1201 and JS-2012 (An et al., 2013; Luo et al., 2014; Gu et al., 2015b; Tong et al., 2015; Ye et al., 2015, 2016; Yang et al., 2016; Zhao et al., 2018). Compared with the Chinese classical strains such as Fa or SC, the PRV variants clearly exhibit increased virulence to pigs from 35 to 127 days old pigs. Pigs infected with PRV variants showed much severer pathological lesions, with extensive antigen distribution in different organs and severe clinical symptoms, such as high fever, respiratory symptoms, and neurological signs (Luo et al., 2014; Tong et al., 2015; Yang et al., 2016).

Genomic characterization of PRV variants revealed their remarkable genetic divergence from European and American strains, including Bartha-K61 (Luo et al., 2014; Ye et al., 2015). Gene deletions, insertions and substitutions are scattered along the genome (Luo et al., 2014; Ye et al., 2015). In this regard, a long-asked question is how the genetic variations contribute to increased viral virulence and escape of host immunity provided by Bartha-K61 vaccine in the field. Progress has recently been made toward to this direction. Yu et al. (2017) have recently shown that the glycoprotein B (gB) contributes to the immunogenic difference between PRV variant JS-2012 and Bartha-K61, and that Bartha-K61 carrying gB from the strain JS-2012 could provide partial protection in a mouse model. While our work is under way, Zhang et al. (2019) showed that Bartha-k61 carrying gD and gC of variant strain AH02LA provided complete clinical protection against the challenge by AH02LA. However, in that study, the piglets immunized with Bartha-K61 did not show any clinical symptom and also all survived the challenge by AH02LA (Zhang et al., 2019). Therefore, it is not clear whether vaccination with the chimeric mutant will protect pigs from lethal challenge of epidemic strains. In addition, the virus-induced lesions, the viral tissue load and the role of individual genes in the protection were not assessed. Thus, there still exists a considerable knowledge gap regarding how the antigenic changes lead to inefficient cross-protection.

In this study, we identified the envelope glycoproteins gB, gC, and gD as the key regions of PRV genetic variation by bioinformatics analysis. By constructing a series of chimeric viruses between Bartha-K61 and epidemic strain HB1201, we found that gC is a major contributor to cross-neutralization against HB1201. Immunization with Bartha-K61 chimera carrying either gC of PRV HB1201 is sufficient to protect piglets from lethal challenge by PRV HB1201 at a lethal dose of 10^7^ TCID_50_. Interestingly, despite a lower cross-neutralization ability, swapping of PRV gD alone also enabled Bartha-K61 to acquire the ability to induce a protective immunity. A better protection in terms of reducing viral load in brain and virus-induced microscopic lesions could be achieved when both gC and gD were swapped. These findings have important implications in development of novel vaccines for PRV control in China.

## Materials and Methods

### Cells, Viruses, and Antibodies

Vero-CCL81 cells were cultured in Dulbecco’s Modified Eagle’s Medium (DMEM) (Gibco, United States) supplemented with 10% fetal bovine serum (FBS) (Gibco, United States). The PRV variant strain HB1201 (Genbank no. KU057086) was isolated from PR outbreak of a swine farm in Hebei province of China in 2012. PRV Bartha-K61 vaccine strain (Genbank no. JF797217.1) was a gift from Dr. Zhijun Tian (Harbin Veterinary Institute, CAAS) preserved in our laboratory. All PRV strains were propagated in Vero-CCL81 cells maintained in DMEM supplemented with 2% FBS (Gibco, United States) at 37°C with 5% CO_2_. The mouse monoclonal antibodies (mAbs) specific to PRV gE were obtained from Beijing Jinnuo Baitai Biotechnology Co., Ltd.

### Construction and Generation of Chimeric PRV Viruses

sgRNAs targeting specific genes were designed using an online CRISPR tool^1^. For each target gene, two sgRNAs were designed that are located on both sides of variation region within the open reading frame. To minimize the off-target effect, we chose those with a high comprehensive score. Following that, the sgRNA oligos were blasted against PRV genome to ensure their high specificity. The sgRNA plasmid of CRISPR/Cas9 was constructed as previously described (Ren et al., 2015). In brief, the oligo pairs were synthesized (Supplementary Table S1) and annealed under the following condition: 5 min at 95°C, 30 min at 25°C. The purified product was then cloned into the plasmid pX335 (sgRNA/Cas9 expression vector) at the restriction site of *Bbs*I followed by verification by DNA sequencing.

To exchange the non-essential gene coding for gC, the homologous arms were amplified from the acceptor strain, and GFP was amplified from the plasmid pEGFP-N2 (Clonetech, CA, Mountain View, United States) by PCR using Phanta^®^ Max Super-Fidelity DNA Polymerase (Vazyme, Nanjing China). The donor GFP flanked by the homologous arms was then generated by overlapping PCR. To make recombinant virus, PRV genome DNA was extracted from the infected Vero-CCL81 cell as previously described (Hirt, 1967), and co-transfected with linear donor DNA (5.0 μg) and two sgRNA plasmids (each 1.5 μg) into Vero CCL81 cells using the lipofectamine^®^ 2000 Reagent (Invitrogen, Carlsbad, CA, United States). The cytopathic effect was monitored daily and the recombinant virus carrying GFP was harvested after 72 h later. The virus was purified by plaque purification with homogeneity monitored by the plaque sizes and confirmed by DNA sequencing. To generate the chimeric gC virus, the GFP was replaced by donor gC flanked by homologous arms from acceptor PRV. The recombinant virus was generated via transfection and screened by loss of GFP fluorescence.

The process for swapping the essential gene is one-step with the procedure very similar to that above for gC. The only difference is that the sites that can be recognized by sgRNA in donor DNA fragment were mutated synonymously to avoid the cleavage of donor sequences by Cas9. All viruses were verified by nucleotide sequencing. The primers used for generating recombinant viruses are included in Supplementary Table S2.

### Multistep Growth Analysis

To analyze the growth property of rescued viruses, Vero-CCL81 cells in 12-well plates were infected with indicated viruses at a multiplicity of infection (MOI) of 0.01. After absorption for 1 h at 37°C, the unbound viruses were inactivated by brief acid wash (135 mM NaCl, 10 mM KCl, 40 mM citric acid, pH 3.0). The acid was then removed by washing the cells with PBS twice, and then the cells were supplemented with fresh DMEM containing 2% FBS. At indicated time points post-infection, the whole culture was harvested; the virus titer was determined by endpoint dilution assay and expressed as 50% tissue culture infectious dose (TCID_50_), according to the Reed-Muench method (Reed and Muench, 1938).

### Neutralization Assay

To perform the virus neutralization assay, anti-Bartha-K61 sera were inactivated at 56°C for 30 min and diluted with DMEM in a series of two-fold dilutions. The serially diluted serum of 50 μL was then mixed with equal volume of 100 TCID_50_ of indicated viruses and incubated at 37°C for 1 h. The mixtures were then used to infect Vero-CCL81 cells in 96-well plates. The virus-induced CPE was examined daily for 72 h following infection and the neutralizing antibody titer was calculated by the Reed-Muench method.

### Animal Experiments

All animal experiments in this study were approved by the Laboratory Animal Ethical Committee of China Agricultural University with the license number (CAU20180823-1). All 4-week-old healthy piglets were confirmed negative for PRRSV, CSFV, PRV and PCV2 by antibodies-based ELISA and PCR.

#### Animal Trial A

Fifteen 4-week-old healthy piglets were divided randomly into three groups with five piglets in each group. The piglets in each group were immunized with either Bartha-K61, Bartha-gC_*HB*__1201_, or Bartha-gD_*HB*__1201_ at a dose of 2 × 10^5^ TCID_50_ via intramuscular (i.m) route. After vaccination, the rectal temperature and clinical symptoms were monitored daily. Serum samples were collected weekly to monitor PRV gB-specific and gE-specific antibody responses. At 28 days post-immunization (dpi), the immunized piglets were challenged with PRV epidemic strain HB1201 at a dose of 2 × 10^7^ TCID_50_ via intranasal route. The clinical signs of disease and rectal temperature were recorded and scored daily. A detailed scoring system is summarized in Supplementary Table S3. At 14 days post-challenge (dpc), all the survived piglets were euthanized and necropsied, and the tissues were collected for viral load, histopathology and immunohistochemistry analyses.

#### Animal Trial B

Eighteen 4-week-old healthy piglets were randomly divided into four groups, including negative control (*n* = 3), unvaccinated group (*n* = 5), Bartha-K61 group (*n* = 5) and Bartha-gCD_*HB*__1201_ group (*n* = 5). Piglets in the vaccinated group were inoculated via intramuscular (i.m) route with 2 × 10^5^ TCID_50_ of either Bartha-K61 or Bartha-gCD_*HB*__1201_, and piglets in unvaccinated group and negative control group were received 2 mL DMEM medium, respectively. Following immunization, the rectal temperature and clinical symptoms were recorded daily. Serum samples were collected weekly to monitor PRV gB-specific and gE-specific antibody responses. At 28 dpi, all pigs were challenged intranasally (i.n) with PRV HB1201 at a dose of 2 mL 10^7^ TCID_50_ except for negative control group. After challenge, the clinical signs of disease and rectal temperature were recorded and scored daily. A detailed scoring system is summarized in Supplementary Table S3. At 14 dpc, all the survived pigs were euthanized and necropsied, and the tissues were collected for viral load, histopathology, and immunohistochemistry (IHC) analyses.

### Quantitative PCR (qPCR)

The viral tissue load was measured by absolute quantitative PCR (qPCR) targeting the gB gene. The viral DNA from tissues were extracted by TIANamp virus DNA/RNA kit (Tiangen, Beijing, China) with the ChamQ^TM^ SYBR^®^ qPCR Master Mix (Vazyme, Nanjing, China) on an ABI 7500 Real-time PCR system (Applied Biosystem, United States) according to the manufacturer’s recommendations. The primers were as follows: upstream primer: 5′-GTCTGTGAAGCGGTTCGTGAT-3′ and downstream primer: 5′-ACAAGTTCAAGGCGCACATCTAC-3′. Seven serial dilutions of plasmid containing gB with the copy number from 10^1^ to 10^7^ copies/μL served as template to generate a standard curve. The PCR was performed in a 20 μL reaction containing 0.4 μL gene specific primers (10 μM), 10 μL ChamQ^TM^ SYBR^®^ qPCR Master Mix (Vazyme, Nanjing China), 2 μL PRV genome, and 7.2 μL ddH_2_O. The PCR parameter was set up as follows: 50°C for 2 min, 95°C for 2 min; 40 cycles of 95°C for 15 s, 60°C for 15 s, and 72°C for 45 s. The viral loads were calculated with the 7500 System SDS software according to standard curve and expressed as log_10_ copies per gram of tissue sample.

### Gross and Histopathological Examinations

At necropsy, the tissues, such as lung, lymph node, kidney, tonsil, and brain, were all assessed for gross lesions. The histopathological examination and IHC were performed as previously described (Halbur et al., 1995, 1996). Briefly, the collected tissues were fixed with 4% paraformaldehyde solution at room temperature for 48 h. The fixed tissues were trimmed, dehydrated in graded alcohol, and embedded in paraffin. Micro sections were cut and stained with hematoxylin and eosin (HE) for microscopic pathological changes. To visualize the antigen load and distribution, the micro sections were also stained with the PRV gE mAb at a dilution of 1:5,000. The severity of lesions was blindly evaluated from 0 to 4 according to previous studies (Halbur et al., 1995, 1996). The IHC scores of PRV antigen were conducted through a range score of 0 to 4 for by calculating the number of gE positive cells.

### Statistical Analysis

Statistical analyses were performed using two-way analysis of variance (ANOVA) test in GraphPad Prism 5 (San Diego, CA, United States). P-values of <0.05 were considered statistically significant; P-values of <0.001 were considered extremely significant.

## Results

### Construction of Chimeric Viruses Between PRV Epidemic Strain HB1201 and the Bartha-K61 Vaccine

**FIGURE 1:**
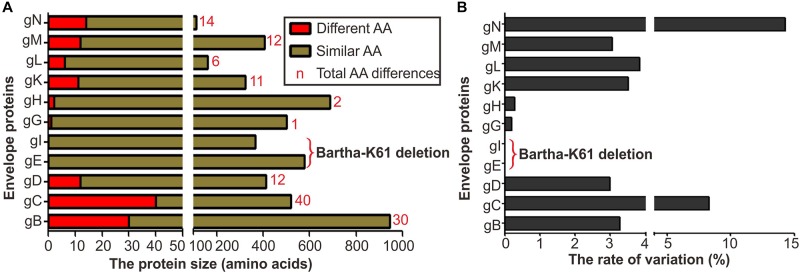
Envelope protein coding variation in PRV variant strain HB1201 versus the vaccine strain Bartha-K61. **(A)** Total number of amino acid differences between HB1201 and Bartha-K61. **(B)** The percentage of amino acid variation in HB1201.

It has been reported that immunization with the Bartha-K61 vaccine does not provide complete protection against challenge of PRV novel variants (An et al., 2013; Luo et al., 2014; Tong et al., 2015). This observation implies that the antigenic changes in viral envelope glycoproteins likely contribute to the escape of either humoral or cellular immunity or both. PRV encodes at least nine envelope glycoproteins, and a sequence comparison between Bartha-K61 and PRV epidemic strain HB1201 (Genbank no: KU057086) showed that gB, gC, gD, gN, gM contain the most large number of mutations (Figure 1). Of note, gB, gC, and gD are the key proteins involved in virus entry and induction of neutralizing antibodies or protective immunity. Thus, our analyses focused on these three proteins.

**FIGURE 2:**
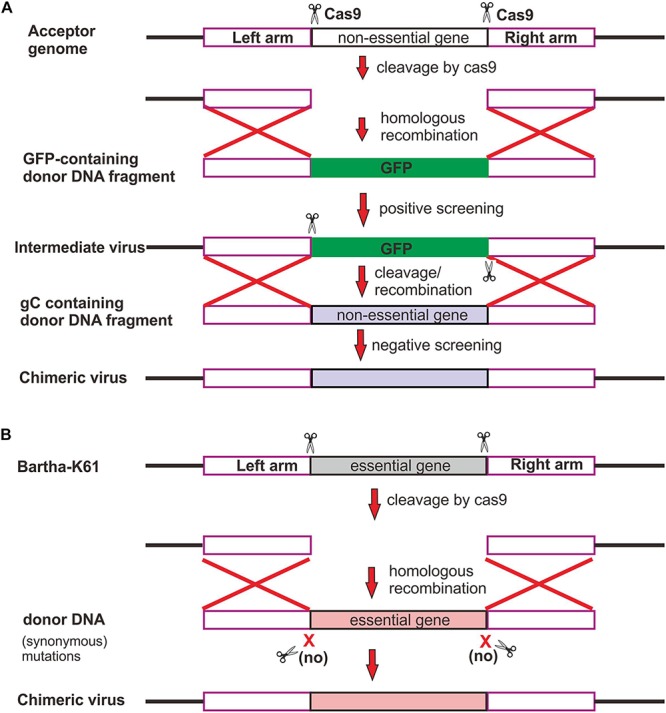
Strategy for constructing PRV chimeric viruses. **(A)** Swapping of non-essential gene. Two sgRNAs were designed to guide Cas9 to cleave off a non-essential gene, and GFP was used for both positive and negative screening of recombinant virus. **(B)** Swapping of essential gene. To increase the rescue rate, Cas9 recognition sites within donor DNA were mutated synonymously to avoid cleavage by Cas9.

**FIGURE 3:**
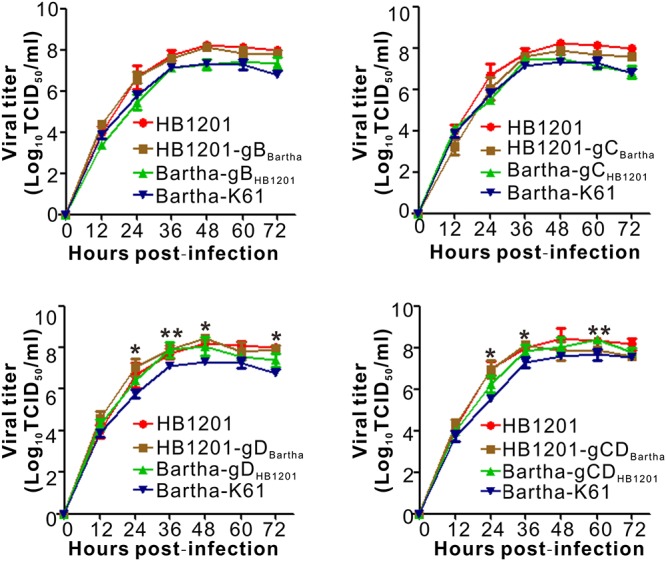
Multistep growth curve of chimeric viruses in Vero cells. Vero-CCL81 cells in 12-well plates were infected with the viruses at an MOI of 0.01. At each indicated time point, total viruses were titrated with the endpoint dilution assay. The data represent means ± standard deviations (SD) of three replicates. Asterisk indicates a significant difference: *P < 0.05; **P < 0.01; ***P < 0.001; NS: no difference.

We assessed the individual contribution of gB, gC, and gD to the neutralizing antibody escape by constructing chimeric mutants via swapping the genes individually or in combination in a reciprocal manner. The chimeric viruses between Bartha-K61 and HB1201 were generated by taking advantage of CRISPR/Cas9 technology and homologous recombination (Figure 2). In brief, CRISPR/Cas9 was used to cleave the target sequence off the virus genome, and the subsequent gene swapping is facilitated via the mechanism of cellular homologous recombination by co-transfecting a gene fragment containing the target gene flanked by homologous arms on both ends (Figure 2). For swapping the non-essential gene coding for gC (Figure 2A), the GFP gene was first inserted into the acceptor genome to facilitate virus purification, which was then replaced in the next round of recombination with the target gene by homologous recombination following cleavage of the target sequence by Cas9. For swapping the essential gene (e.g., gB and gD) (Figure 2B), the donor sequence was designed to contain synonymous mutations to avoid cleavage by Cas9. By using these strategies, a total of eight chimeric viruses were made, including HB1201-gB_*Bartha*_ and Bartha-gB_*HB*__1201_; HB1201-gC_*Bartha*_ and Bartha-gC_*HB*__1201_; HB1201-gD_*Bartha*_ and Bartha-gD_*HB*__1201_; HB1201-gCD_*Bartha*_ and Bartha-gCD_*HB*__1201_. It should be noted that HB1201-gCD_*Bartha*_ and Bartha-gCD_*HB*__1201_ were constructed based on HB1201-gC_*Bartha*_ and Bartha-gC_*HB*__1201_, respectively. After 3–4 rounds of plaque purification and sequencing verification, homogeneous viruses were obtained as assessed by plaque purification. The multi-step growth curve analyses revealed that the growth kinetics of gB and gC recombinant viruses was generally similar to their respective parental viruses, and the virus titer of Bartha-gD_*HB*__1201_ and Bartha-gCD_*HB*__1201_ was slightly higher than Bartha-K61 at some time points (*P* < 0.05) (Figure 3).

### gC Is a Major Contributor to Inefficient Cross-Neutralization Against HB1201

**FIGURE 4:**
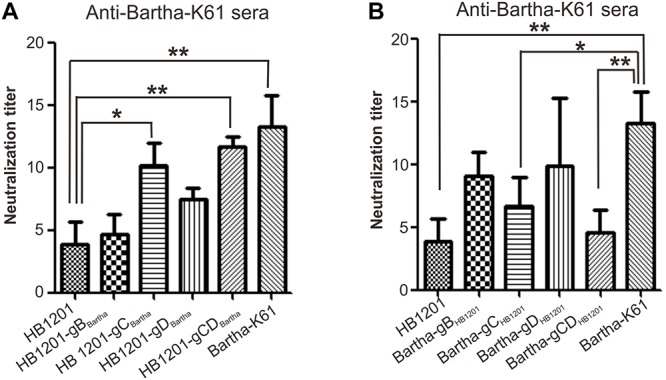
Sensitivity of chimeric viruses to swine neutralizing antibodies to Bartha-K61. **(A)** Sensitivity of PRV strain HB1201 and its derivatives to anti-Bartha-K61 sera. **(B)** Sensitivity of PRV Bartha-K61 and its derivatives to anti-Bartha-K61 serum. Virus neutralization assays were performed with antisera from three individual piglets. The data represent means ± standard deviations (SD) of three replicates. Asterisk indicates a significant difference: *P < 0.05; **P < 0.01; ***P < 0.001; NS: no difference.

The sensitivity of chimeric viruses to neutralizing antibodies was investigated by using swine anti-Bartha-K61 serum (Figure 4). As expected, anti-Bartha-K61 serum had a much higher neutralization titer (NT) against Bartha-K61 than HB1201, and a difference of about threefold could be discerned. In the gain-of-function test, swapping Bartha-K61 gC (HB1201-gC_*Bartha*_) enabled HB1201 gained the sensitivity to anti-Bartha-K61 serum (Figure 4A), while the mutant HB1201-gD_*Bartha*_ is less sensitive to the neutralizing antibodies (Figure 4A). Moreover, swapping of both gC and gD had a similar effect to gC alone, suggesting that variability of gC plays a major role in the neutralization escape. Interestingly, swapping of gB (HB1201-gB_*Bartha*_) did not have a statistically significant effect on the sensitivity of neutralization (Figure 4A). In the loss-of-function test (Figure 4B), anti-Bartha-K61 serum had significantly reduced NT against Bartha-gC_*HB*__1201_, and the lowest occurred to Bartha-gCD_*HB*__1201_, a titer that was comparable to HB1201. Interestingly, swapping of only gD (Bartha-gD_*HB*__1201_) did not have an effect that was statistically significant. Together, these results suggest that gC is a major contributor to the inefficient cross-neutralization *in vitro*.

### Substitution of Either gC or gD Alone Can Enable Bartha-K61 to Induce Protective Pig Immunity Against Lethal Challenge by Epidemic Strain HB1201

Considering the individual contribution to the escape of neutralizing antibodies, we proceeded with the chimeric viruses Bartha-gC_*HB*__1201_ and Bartha-gD_*HB*__1201_ to assess their ability to induce a protective immunity. One-month-old piglets were immunized the piglets with either Bartha-gC_*HB*__1201_, Bartha-gD_*HB*__1201_, or Bartha-K61 via intramuscular route with a dose of 10^5^ TCID_50_. All piglets survived, had similar daily gain, and did not exhibit any apparent clinical symptom (Supplementary Figure S1), suggesting that these chimeric mutants are avirulent to piglets. We also measured the antibody induction following immunization (Supplementary Figure S2). The serum samples were collected at weekly internals, and the levels of the gB and gE antibodies were measured by PRV blocking ELISA. The S/N ratio of gB antibodies decreased slightly at 7 dpi. At 14 dpi, the gB-specific antibodies were detected in all immunized pigs (Supplementary Figure S2). In contrast, there were no detectable antibodies to gE.

**FIGURE 5:**
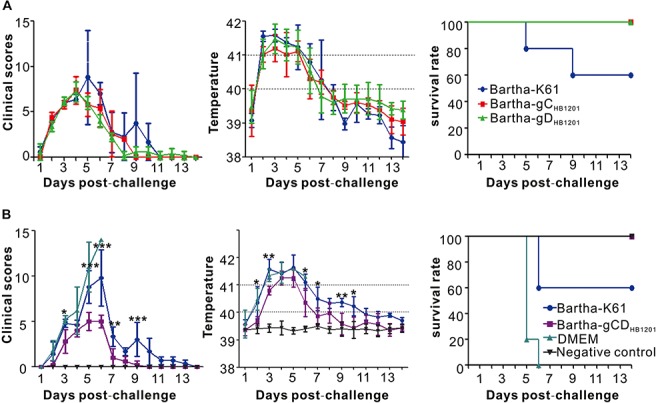
Protective efficacy of chimeric virus-induced immunity against the lethal challenge by PRV epidemic strain HB1201. **(A)** Clinical scores, rectal temperature, and survival rate of each group piglets immunized after HB1201 lethal challenge. **(B)** The same as **(A)** except that Bartha-K61 and Bartha-gCD_*HB*__1201_ were used for immunization. Data are presented as mean ± SD and asterisk indicates a significant difference between Bartha-gCD_*HB*__1201_ and Bartha-K61 in clinical scores and the rectal temperatures. *P < 0.05; **P < 0.01; ***P < 0.001.

At 28 days post-immunization, the piglets were challenged with PRV epidemic strain HB1201 via nasal route with a highly lethal dose of 10^7^ TCID_50_. Following challenge, all piglets developed fever beginning at 2 dpc and reached the peak at 3–4 dpc with rectal temperatures as high as 40.0–42°C. The three immunized groups (Bartha-gC_*HB*__1201_, Bartha-gD_*HB*__1201_, and Bartha-K61) did not exhibit a statistically significant difference in temperature fluctuation. However, clinically, piglets in the Bartha-K61 group showed significant respiratory symptom, loss of appetite, depression, vomiting, convulsion, and ataxia. Moreover, two piglets showed significant CNS symptoms and died at 5 and 9 dpc, respectively (Figure 5A). In contrast, all piglets in other two groups survived the challenge, and none showed any apparent CNS symptom throughout the study, suggesting that Bartha-gC_*HB*__1201_ and Bartha-gD_*HB*__1201_ induced protective immunity.

**FIGURE 6:**
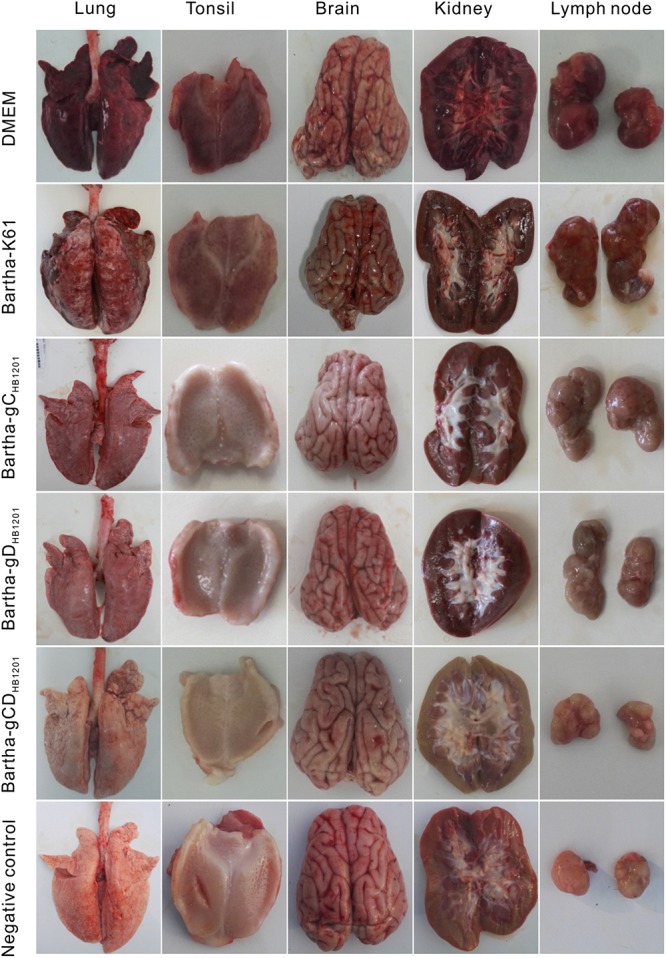
Gross lesion changes of immunized pigs following challenge with PRV strain HB1201. Different tissues of the piglets (lung, tonsil, brain, kidney, and submandibular lymph nodes) were collected and subjected to pathological examination at 14 days post-challenge (dpc) with representative gross lesions shown here.

At 14 dpc, the survived piglets were euthanized and subjected to necropsy for pathological examination. For the Bartha-K61 group, the two piglets that died following the challenge showed severe hemorrhage in lung, lymph nodes and kidney; pulmonary consolidation; and brain edema. For the piglets immunized with Bartha-gC_*HB*__1201_ and Bartha-gD_*HB*__1201_, moderate or mild hemorrhagic and consolidation lesions were observed in lungs and only light swelling in lymph nodes. We did not observe visible pathological lesions in brains and tonsils (Figure 6). The overall assessment was shown in Supplementary Table S4.

**FIGURE 7:**
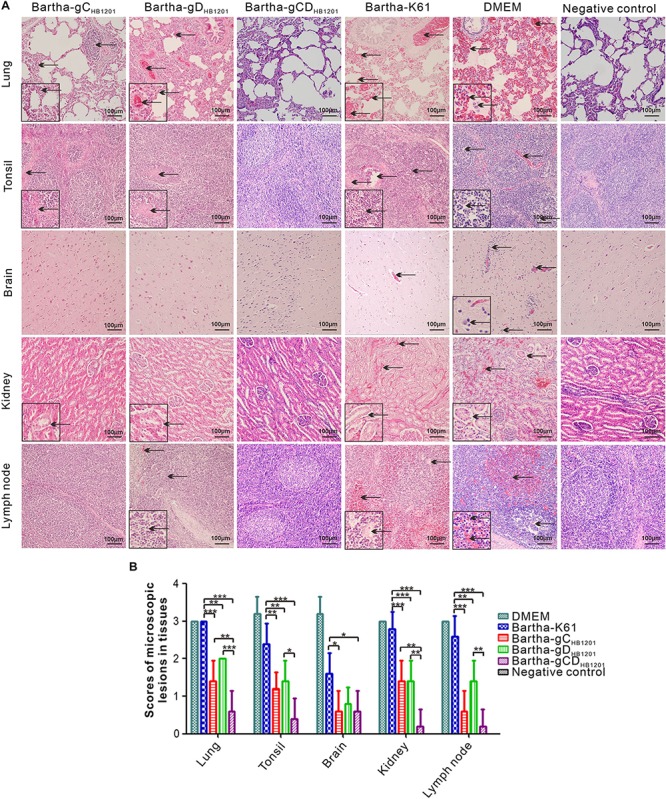
Histopathological lesions immunized pigs following challenge with PRV strain HB1201. Different tissues as indicated were fixed, sectioned, and stained with hematoxylin and eosin (HE). **(A)** The representative tissue lesions of each group following challenge (HE staining, 200× magnification). **(B)** The quantitative analyses of the tissue lesions. Asterisk indicates a significant difference, *P < 0.05; **P < 0.01; ***P < 0.001.

We also looked into the histopathological changes by HE staining. As shown in Figure 7A, the Bartha-K61 group developed severe microscopic lesions of multiple organs, such as alveolar septal capillary dilatation, hemorrhage, congestion, alveoli disappearance; tonsil necrosis and congestion; lymphocyte infiltration around the blood vessels or nerve cells in brain; severe hemorrhage and cortical necrosis in submandibular lymph nodes; and renal tubular epithelial cells detachment in kidney. In contrast, Bartha-gC_*HB*__1201_ and Bartha-gD_*HB*__1201_ groups exhibited moderate histopathological changes in lung, tonsil, kidney, and submandibular lymph nodes, and no apparent histopathological changes were found in brain (Figure 7A). Correspondingly, the microscopic lesion scores in all tissues of these two groups were significantly lower than that in Bartha-K61-immunized group (Figure 7B). Thus, Bartha-gC_*HB*__1201_ and Bartha-gD_*HB*__1201_ provide much better protection of piglets from microscopic injuries.

### Immunization With Bartha-gC_*HB*__1201_ or Bartha-gD_*HB*__1201_ Can Significantly Reduce Viral Tissue Load but Not Brain

**FIGURE 8:**
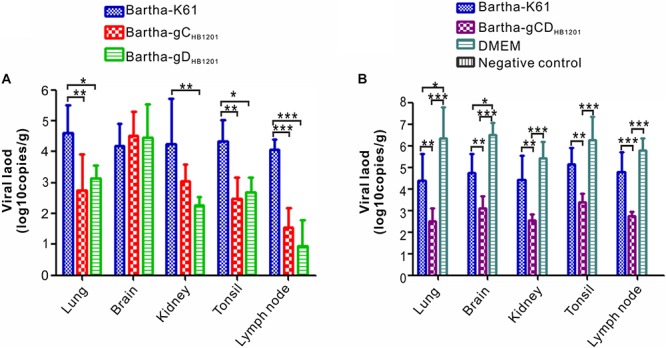
The viral tissue load of PRV HB1201 by qPCR. **(A)** Viral tissue load of the pig groups immunized with Bartha-K61, Bartha-gC_*HB*__1201_, or Bartha-gD_*HB*__1201_. **(B)** Viral tissue load of the groups immunized with Bartha-K61, Bartha-gCD_*HB*__1201_ or DMEM after HB1201 challenge. Asterisk indicates a significant difference: *P < 0.05; **P < 0.01; ***P < 0.001.

**FIGURE 9:**
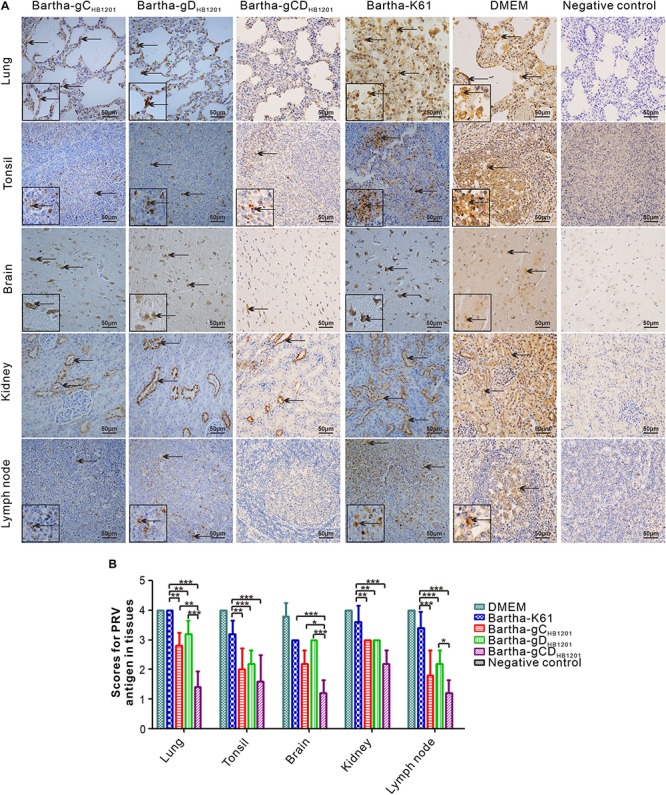
Immunohistochemistry analysis of viral tissue distribution. **(A)** Different tissues as indicated were fixed, sectioned, and stained with mouse monoclonal antibodies (1:500) to PRV gE, and the arrows show the positive signals for PRV in the tissues (400× magnification). **(B)** The IHC scores of PRV antigen were analyzed by calculating the number of gE positive positive cells. Asterisk indicates a significant difference, *P < 0.05; **P < 0.01; ***P < 0.001.

The viral load in the tissues of pigs challenged PRV HB1201 were assessed using quantitative real-time PCR with primers targeting the gene coding for gB. The results showed that immunization with Bartha-gC_*HB*__1201_ or Bartha-gD_*HB*__1201_ significantly reduced viral tissue load in lung, kidney, tonsil, and lymph nodes by several logs compared with Bartha-K61 group (Figure 8A). However, the difference in brain was not statistically significant. Accordingly, the scores of PRV antigen in organs of the piglets vaccinated with Bartha-gC_*HB*__1201_ and Bartha-gD_*HB*__1201_ were much lower than the group Bartha-K61 with the exception of brain (Figure 9B). For the negative control group, PRV antigens were not detectable in all piglets. For the DMEM group, the PRV antigens were distributed in all the organs with specific signals detected in alveolar epithelial cells of lung, nerve cells of brain, renal tubular epithelial cells, and lymphatic nodules of tonsils and submandibular lymph nodes (Figure 9).

### Bartha-K61 Carrying Both gC and gD of PRV Strain HB1201 Provides Better Protection and Significantly Reduces Viral Load in Brain

We next investigated whether Bartha-gCD_*HB*__1201_ can provide a better protection than Bartha-gC_*HB*__1201_ or Bartha-gD_*HB*__1201_ alone. To this end, piglets were immunized with Bartha-K61, Bartha-gCD_*HB*__1201_, or DMEM. At 28 dpi, the piglets of all groups were challenged with a lethal dose of PRV HB1201 (10^7^ TCID_50_), and the clinical signs were monitored and scored (Figure 5A). For the non-vaccinated group (DMEM), the pigs displayed steady progression of PR syndrome, from fever to respiratory and CNS symptoms, and all died within 6 dpc. For the Bartha-K61 group, the piglets also showed significant respiratory symptoms, depression, convulsion signs and ataxia, and two of them died at 6 dpc exhibiting CNS symptoms such as ataxia and agitation. In contrast, the Bartha-gCD_*HB*__1201_ group showed only transient, slight loss of appetite and respiratory symptom between the third and fifth days. However, the piglets in this group developed fever, which lasted for 4 days before returning back to normal, but this duration was much shorter that Bartha-K61 group, which lasted for 6 days. In addition, the Bartha-gCD_*HB*__1201_ group had generally much lower rectal temperatures than the Bartha-K61 group at different days post challenge.

Post-mortem necropsy did not find obvious gross lesions in all organs immunized with Bartha-gCD_*HB*__1201_ (Figure 6 and Supplementary Table S5). Consistently, the HE staining revealed significantly reduced microscopic lesions in all tissues examined (Figure 7). For the viral tissue load (Figure 8B), Bartha-gCD_*HB*__1201_ significantly reduced the viral load in brain, in addition to other tissues, a stark difference from that for the groups Bartha-gC_*HB*__1201_ and Bartha-gD_*HB*__1201_. Immunohistochemical staining of viral antigens showed the similar trend (Figure 9). Thus, gC and gD display apparently a synergistic effect on induction of protective immunity against lethal challenge of epidemic strain HB1201.

## Discussion

The PRV epidemic strains can escape from the immunity provided by the classical Bartha-K61 vaccine, but the underlying mechanisms have remained poorly understood. By constructing chimeric viruses, here we revealed the following findings: (i) gC was a critical contributor to the inefficient cross-neutralization ability to epidemic strain; (ii) swapping of either gC or gD alone enabled Bartha-K61 to induce a protective immunity against the lethal challenge by PRV strain HB1201; and (iii) gC and gD displayed a synergistic effect, and immunization with Bartha-K61 carrying both gC and gD of HB1201 could significantly decrease viral load in brain and provide much better protection. The relevant significance or insights of this study are discussed below.

The glycoproteins gB, gC, and gD are involved in virus entry and are critical targets of neutralizing antibodies (Mettenleiter, 1996; Gerdts et al., 1997, 1999; Pomeranz et al., 2005). In particular, gC mediates the initial attachment of PRV to cell surface. It acts through distinct viral heparin-binding domains (HBDs) that are located in its N-terminal region (Flynn and Ryan, 1996; Ober et al., 2000), and consequently, antibodies targeting this antigenic domain have been shown to interfere with the viral attachment (Ober et al., 2000). In addition, a large portion of the neutralizing activity of pooled convalescent swine sera have been found to be directed against gC (Ben-Porat et al., 1986). Thus, it is not very surprising that gC shows a high level of genetic variability among viral strains across different geographic regions. Our studies show that gC has the highest mutation rate and that the genetic mutations are mainly scattered in the N-terminal region between 14 aa and 243 aa. Further, we provide evidence to show that gC variability is a key factor for the low capacity of anti-Bartha-K61 serum to neutralize the epidemic strain HB1201 (Figure 4). Future studies may be directed to map critical amino acids for the antigenic difference. On the other hand, gB and gD are important for virus penetration (Pomeranz et al., 2005). Our analyses showed that the genetic variation of gD is a secondary factor for the inefficient cross-neutralization (Figure 4). Despite a lower cross-neutralization ability, swapping of gD from PRV epidemic strain HB1201 enabled Bartha-K61 to acquire the ability to induce a protective immunity, suggesting a possibility of gD-induced cellular immunity in the cross-protection. The gain or loss-of-function tests also revealed a less critical role in cross-neutralization for gB variability (Figure 4). Interestingly, Yu et al. (2017) have recently shown that Bartha-K61 carrying gB from the strain JS-2012 could provide partial protection in a mouse model. It will be worthy to test whether the corresponding virus can also induce a protective immunity in piglets.

Our studies also provide a glimpse into the mechanisms of viral clearance in central nervous system. It is noticeable that neither Bartha-gC_*HB*__1201_ nor Bartha-gD_*HB*__1201_ was able to reduce viral load in brain, although either gC or gD-substituted mutants was capable of inducing protective immunity (Figure 8A). In contrast, simultaneous substitution of both provided much better protection. There were no obvious or significantly reduced microscopic lesions revealed by HE staining in all organs immunized with Bartha-gCD_*HB*__1201_ (Figure 7). Moreover, this significantly reduced the viral load in brain (Figure 8B). Collectively, these results suggest that the clearance of PRV from brain may require immunity induced by multiple viral antigens. Previous studies have demonstrated that neutralizing antibodies play an important role in mediating clearance of several neurotropic viruses from central nervous system, such as rabies virus, sindbis virus, murine hepatitis virus, and so on (Dietzschold et al., 1992; Matthews et al., 2001; Burdeinick-Kerr et al., 2007; Hooper et al., 2009). Emerging evidence indicates that antibody blockade of neurotropic viruses requires CD4^+^ T cell-dependent opening of the blood–brain barrier (BBB). For example, antibody-dependent protection against HSV-2 requires IFN-γ secretion by CD4^+^ T cells in the mouse model (Iijima and Iwasaki, 2016), and CD4^+^ T cell-dependent antibody access to the CNS is likely required for protection against Rabies virus (Hooper et al., 1998). Since the gD genetic variability contributes secondarily to inefficient cross-neutralization but the corresponding chimeric mutant was capable of inducing protective immunity, we speculate that gD-induced cellular immunity may have a cooperative role together with the neutralizing antibodies in helping reduce the viral load in brain. Further experiments are required to test this hypothesis in the future.

Our studies also showed that Bartha-gCD_*HB*__1201_ is sufficient to induce an immunity against the lethal challenge at a high dose of 10^7^ TCID_50_, a dose that is 10 to 100 times higher than that used normally (Wang et al., 2014; Gu et al., 2015a; Cong et al., 2016; Tong et al., 2016). It should be noted that although the mutant could alleviate the clinical symptoms rather significantly, but it did not stop piglets from developing a transient fever (Figure 5B). Consistent with our studies, Hu et al. (2015) found that piglets vaccinated with rSMXgI/gEΔTK attenuated strain with a dose of 10^6^ TCID_50_ displayed fever lasting 4 days with rectal temperature between 40°C and 42°C after challenge with 10^7^ TCID_50_ of PRV variant SMX strain. Similar results were found in another study based on inactivated ZJ01ΔgE/gI vaccine (Gu et al., 2015a). In contrast, PRV HN1201 TK^–^/gE^–^/gI^–^ strain could provide full protection for young piglets against challenge with HN1201, and no clinical symptom and increasing temperature were observed (Zhang et al., 2015). It is noteworthy that in that study the immunization dose is the same as the challenge dose; both are 10^7^ TCID_50_. In another study, Tong et al. (2016) used PRV variant JS-2012 derivative JS-2012-ΔgE/gI for vaccination at a dose of 10^5^ TCID_50_ and JS-2012 for challenge with a dose of 10^5^ TCID_50_. Similarly, no fever was developed. Since the immune responses are usually dose-dependent, we speculate that the clinical protective effect may be contingent to the dose used; a high challenge dose may overwhelm the host immune system, leading to fever development. Future studies should be focused on dose-dependent immunization and challenge experiment to determine the most appropriate dose for immunization and highest dose an immunized pig can resist.

CRISPR/Cas9 system is a powerful tool for targeted and precise genome editing in eukaryotic cells (Cong et al., 2013). So far, it has been widely used in genome editing of many viruses, such as herpes simplex virus (Lin et al., 2016; Roehm et al., 2016), adenovirus (Bi et al., 2014), hepatitis B virus (Liu et al., 2018), African swine fever virus (Borca et al., 2018; Hubner et al., 2018), and so on. However, most of manipulations were focuses on viral non-essential genes and on insertion of a foreign gene (Xu et al., 2015; Guo et al., 2016; Liang et al., 2016; Tang et al., 2016, 2017, 2018). There is no good approach to replace essential genes among the different PRV strains. The key dilemma is that Cas9 can cleave donor sequence, in addition to the target gene, preventing efficient rescue of the recombinant viruses. We developed a novel strategy to solve this problem (Figure 2). That is, the sgRNA-targeted donor region was mutated by synonymous mutations to avoid the cleavage by Cas9. In this sense, only recombined virus could be rescued because of essentiality of the target gene. We also found that the length of homologous arm is important for recombination. Although previous study indicated that homologous sequences (50 bp) could result in a higher recombination (Zheng et al., 2014), we found that longer homologous arms of about 0.5 kb could increase the efficiency of homologous recombination, leading to higher efficiency of virus rescue. In addition, we found that donor DNA in linear form was more efficient in homologous recombination than the circular form. Together, our improvement of CRISPR/Cas9 platform provides an important means for manipulating essential viral genes in the future. This should aid rapid generation of recombinant PRV viruses for vaccine development and dissection of the function of essential genes.

## Data Availability Statement

All datasets generated for this study are included in the article/Supplementary Material.

## Ethics Statement

The animal study was reviewed and approved by the Laboratory Animal Ethical Committee of China Agricultural University.

## Author Contributions

JH and HY conceptualized the experiments. JR and HW developed the methods and performed the investigation and data validation. LZ, XNG, and XG contributed reagents and analyze the data. JR drafted the original manuscript. JH revised the manuscript.

## Conflict of Interest

The authors declare that the research was conducted in the absence of any commercial or financial relationships that could be construed as a potential conflict of interest.

## Footnotes

1 http://crispr.mit.edu/
