# Supplementary figures and images for: Glycoproteins C and D of PRV Strain HB1201 Contribute Individually to the Escape From Bartha-K61 Vaccine-Induced Immunity
Source: Front Microbiol. 2020 Mar 10;11:323. doi: 10.3389/fmicb.2020.00323 (PMC7076175; doi:10.3389/fmicb.2020.00323)

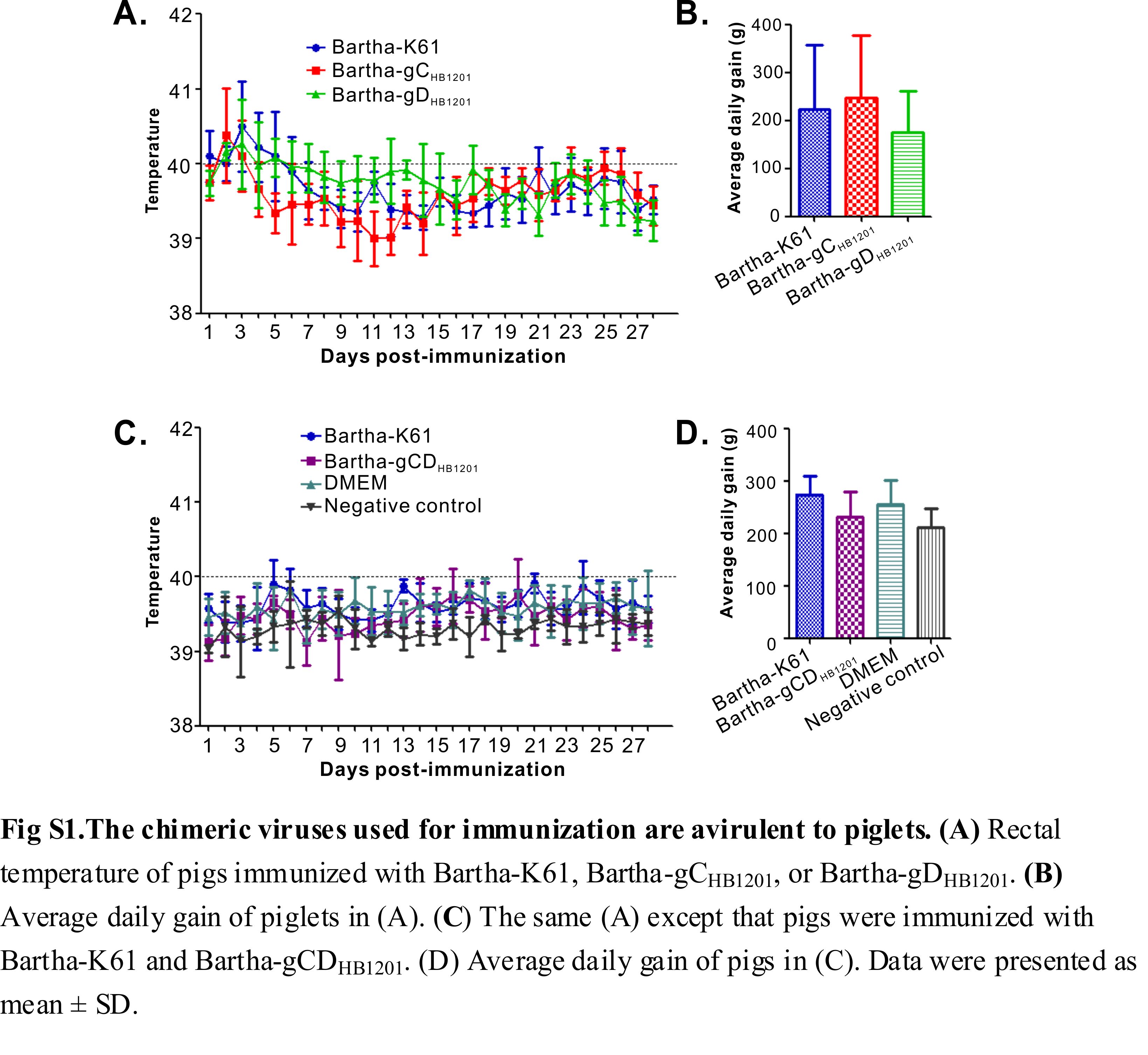

Supplement: Supplementary file 1 [file Image_1.JPEG]

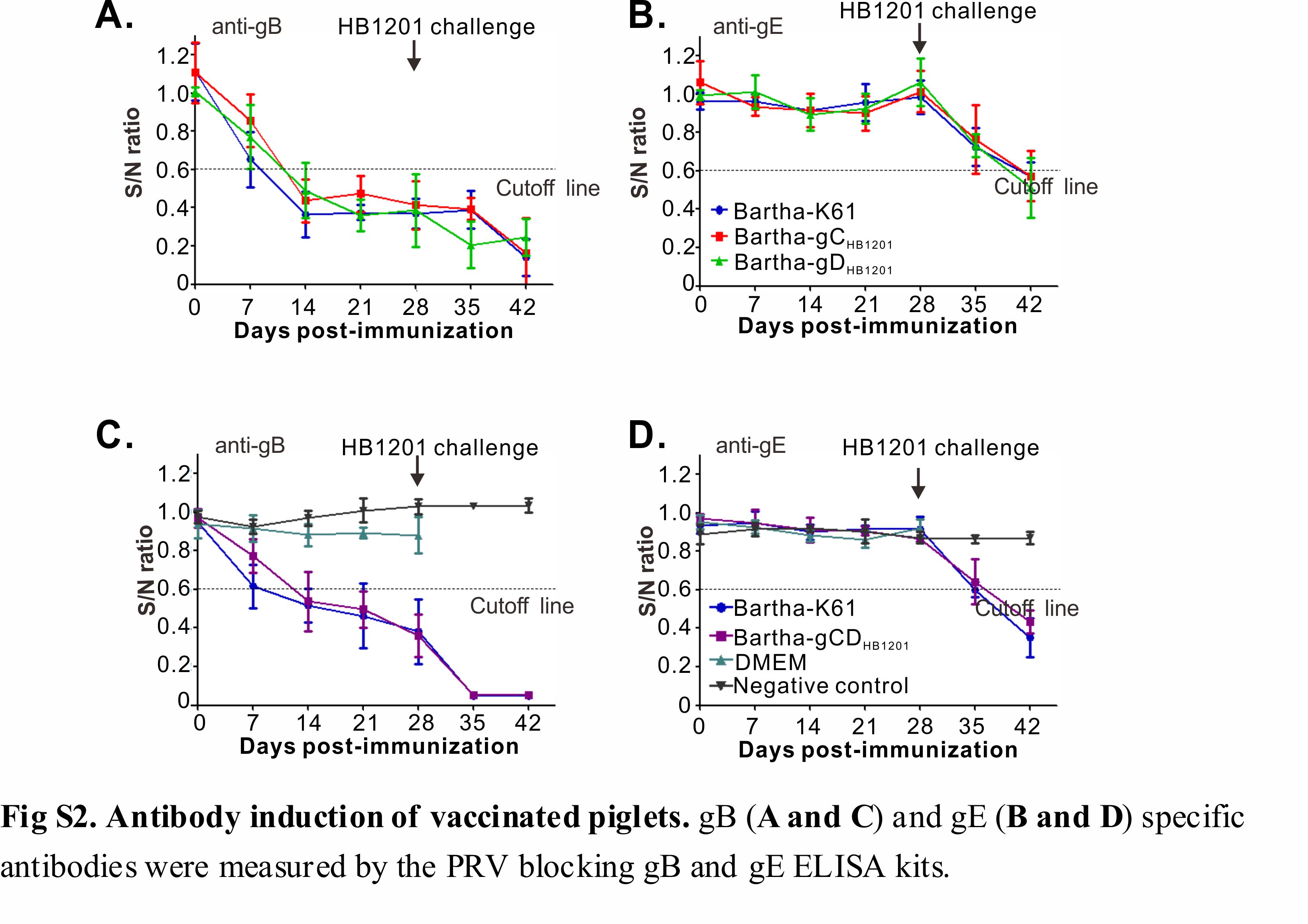

Supplement: Supplementary file 2 [file Image_2.JPEG]
