# Supplementary material for: Glycoproteins C and D of PRV Strain HB1201 Contribute Individually to the Escape From Bartha-K61 Vaccine-Induced Immunity
Source: Front Microbiol. 2020 Mar 10;11:323. doi: 10.3389/fmicb.2020.00323 (PMC7076175; doi:10.3389/fmicb.2020.00323)
Supplement: Supplementary file 3 [file Table_1.DOCX]

Supplementary Material

**Table S1 oligos to construct the sgRNA plasmid used in this study**.

| sgRNA oligos | sequences(5’-3’) |
| --- | --- |
| gB-left-sgRNA-F | CACCGGTCAAGGTGGACCACAACG |
| gB-left-sgRNA-R | AAACCGTTGTGGTCCACCTTGACC |
| gB-right-sgRNA-F | CACCGTGCCCGGGCCGATGCCCGC |
| gB-right-sgRNA-R | AAACGCGGGCATCGGCCCGGGCAC |
| gC-left-sgRNA-F | CACCGAGCATCGCACGCGCGAGCG |
| gC-left-sgRNA-R | AAACCGCTCGCGCGTGCGATGCTC |
| gC-right-sgRNA-F | CACCCGACGTGCGTCTACTACCGC |
| gC-right-sgRNA-R | AAACGCGGTAGTAGACGCACGTCG |
| GFP-left-sgRNA-F | CACCGCCCTTGCTCACCATGAGCG |
| GFP-left-sgRNA-R | AAACCGCTCATGGTGAGCAAGGGC |
| GFP-right-sgRNA-F | CACCTGGACGAGCTGTACAAGCGC |
| GFP-right-sgRNA-R | AAACGCGCTTGTACAGCTCGTCCA |
| gD-left-sgRNA-F | CACCGACTCGGTGTACGGGTACGC |
| gD-left-sgRNA-R | AAACGCGTACCCGTACACCGAGTC |
| gD-right-sgRNA-F | CACCGTCCGTAGCCTCCGCAGTAC |
| gD-right-sgRNA-R | AAACGTACTGCGGAGGCTACGGAC |
