# Supplementary material for: Glycoproteins C and D of PRV Strain HB1201 Contribute Individually to the Escape From Bartha-K61 Vaccine-Induced Immunity
Source: Front Microbiol. 2020 Mar 10;11:323. doi: 10.3389/fmicb.2020.00323 (PMC7076175; doi:10.3389/fmicb.2020.00323)
Supplement: Supplementary file 4 [file Table_2.DOCX]

Supplementary Material

**Table S2 Primers for amplification of genes in this study.**

| Primers | Sequences(5’-3’) |
| --- | --- |
| gB-left arm-F | CTASRGGGCGTCGGGGTCCTCGTTCTC |
| gB-left arm -R | GACCGCGTAGTAAAAGTAGATCATAATGTGGTGCTGCTGCGCGGCATCGCCAACTTCT |
| gB-right arm-F | ATGTCCTGGTCGGTGTCCTCGGGGCCCGCGCCAAAGACCGCCACCAG |
| gB-right arm -R | ACGGGCTGCTTCGACGCGCACAGCTTCATG |
| gB-F: | CACATTATGATCTACTTTTACTACGCGGTCRATGTCGTAGAACTTGAG |
| gB-R: | CGCGGGCCCCGAGGACACCGACCAGGACATCACGGCGGTGCTGGCCTCGGACGTCTTTG |
| HB-gC-left arm-F | CCACGTCTACGCCGGCAGCATCGC |
| HB-gC-left arm -R | GGCGCGTGCGAATGGCCCCCCGTC |
| HBgC-right arm-F | CGTGCTGGTCATCATGGCGACGTGCGTCTACTACCGCCGG |
| HB-gC-right arm -R | CCGGCTCCAGCAGCGGCGGCCGCC |
| HB-GFP-F | TGGGGGGACGGGGGGCCATTCGCACGCGCCATGGCCTCGCTCATGGTGAGCAAGGGCGAGGAGCTG |
| HB-GFP-R | CTTGTACAGCTCGTCCATGCCGAG |
| HB-gC-F | GGGGGCCATTCGCACGCGCCATGGCCTCGCTCGCGCGTGCGATGCTCGC |
| HB-gC-R | ACGCGCGGGGCGTCACGGCCCCGCCCGGCGGTAGTAGACGCACGTCGCCATGA |
| Bartha-gC-left arm-F | GCTCTTCGCCCTCGTCTTATCGGCCGCCTC |
| Bartha-gC-left arm -R | GCGAGGCCATGGCGCGTGCGAATGGCCCCC |
| Bartha-gC-right arm-F | CGCCGGGCGGGGCCGTGACGCCCCGCGCGT |
| Bartha-gC-right arm -R | CAGCCGGTGGCCGTGCCCGCCGCCGCCGCG |
| Bartha-GFP-F | GGGGGCCATTCGCACGCGCCATGGCCTCGCTCATGGTGAGCAAGGGCGAGGAGCTG |
| Bartha-GFP-R | ACGCGCGGGGCGTCACGGCCCCGCCCGGCGCTTGTACAGCTCGTCCATGCCGAGAGTGAT |
| Bartha-gC-F | TGGGGGGACGGGGGGCCATTCGCACGCGCCATGGCCTCGCTCGCGCGTGCGATGC |
| Bartha-gC-R | CCGGCGGTAGTAGACGCACGTCGCCATGAT |
| gD-left arm-F | CGGCCTCCAGCTCTCGGTCGAGACCGAGAC |
| gD-left arm -R | CAGCTGCCACGATTCTGTATATGGATATGCGGGCGGGGGGAAGGTCGGCGCGGGCAC |
| gD-right arm-F | GACCATAAGGAGGGGTCTACCGGCGTCGATGATGATGGTGG |
| gD-right arm -R | GCAGCACGTACGACCCCGCGTCCCCCGAGG |
| gD-F: | GCATATCCATATACAGAATCGTGGCAGCTGACGCTGACGACGGTCCCC |
| gD-R: | ATCGACGCCGGTAGACCCCTCCTTATGGTCCGGGCTGCGCTTTTAGCTCGTCGGCGTCCG |

The base marked in red represents the base of synonymous mutation. R represents A or G.
