# Supplementary material for: Glycoproteins C and D of PRV Strain HB1201 Contribute Individually to the Escape From Bartha-K61 Vaccine-Induced Immunity
Source: Front Microbiol. 2020 Mar 10;11:323. doi: 10.3389/fmicb.2020.00323 (PMC7076175; doi:10.3389/fmicb.2020.00323)
Supplement: Supplementary file 5 [file Table_3.DOCX]

Supplementary Material

**Table S3 Clinical signs scoring system used for analyzing immune protective efficacy of chimeric viruses**

|  | Clinical signs | Criteria [for](app:ds:for) [evaluation](app:ds:evaluation) | Score |
| --- | --- | --- | --- |
| Gross clinical score (GCS) | ①Body temperature | T ≤39.9℃ | 0 |
|  |  | 40.0℃≤T≤40.9℃ | 1 |
|  |  | 41.0℃≤T | 2 |
|  | ②Appetite | Normal | 0 |
|  |  | Inappetence | 1 |
|  | ③Awareness | Normal | 0 |
|  |  | Depression | 1 |
|  | ④Skin | Normal | 0 |
|  |  | Rubefaction | 1 |
| Respiratory clinical score (RCS) | ①Respiratory condition | Normal | 0 |
|  |  | Tachypnea (nervous) | 1 |
|  |  | Tachypnea (calm) | 2 |
|  |  | Tachypnea and dyspnea | 3 |
|  |  | Tachypnea, dyspnea and Irregular respiration | 4 |
|  | ②Cough | Normal | 0 |
|  |  | Cough | 1 |
|  | ③Rhinorrhoea | Normal | 0 |
|  |  | Rhinorrhoea | 1 |
| Nervous signs score (NSS) | ①Nervous signs | Normal | 0 |
|  |  | Shiver | 1 |
|  |  | Ataxia | 2 |
|  |  | Limbs paddling | 3 |
|  |  | Paralysis | 4 |

Usual condition: total score = GCS+RCS+NSS

If piglet died: total score = GCS+RCS+NSS+5

0 ≤ total score ≤ 20
