# Supplementary material for: Glycoproteins C and D of PRV Strain HB1201 Contribute Individually to the Escape From Bartha-K61 Vaccine-Induced Immunity
Source: Front Microbiol. 2020 Mar 10;11:323. doi: 10.3389/fmicb.2020.00323 (PMC7076175; doi:10.3389/fmicb.2020.00323)
Supplement: Supplementary file 7 [file Table_5.DOCX]

Supplementary Material

**Table S5 Pathological changes of pigs immunized with Bartha-gCD_HB1201_ after challenged with the PRV HB1201 strain.**

| Organs | Pathological changes | DMEM | Bartha-K61  (lesion rate) | Bartha-gCD_HB1201_  (lesion rate) | Negative control |
| --- | --- | --- | --- | --- | --- |
| Lung | consolidation | +++ (5/5) | +++ (2/5), + (3/5) | + (5/5) | - |
|  | swelling | +++ (5/5) | +++ (2/5), ++ (2/5), + (1/5) | - | - |
|  | congestion | +++ (5/5) | +++ (2/5), ++ (2/5), + (1/5) | - | - |
|  | hemorrhage | +++ (5/5) | +++ (2/5) | - | - |
| Tonsil | hemorrhage | +++ (2/5), ++ (1/5) | ++ (2/5) | - | - |
|  | congestion | +++ (2/5), ++ (2/5), + (1/5) | +++ (2/5), + (1/5) | - | - |
| Brain | hemorrhage | +++ (1/5), ++ (2/5) | ++ (2/5), + (1/5) | - | - |
|  | congestion | +++ (2/5), ++ (2/5), + (1/5) | ++ (2/5) | - | - |
| kidney | hemorrhage | +++ (5/5) | +++ (3/5), ++ (1/5) | ++ (1/5) | - |
| submandibular Lymph node | hemorrhage | +++ (3/5), ++ (2/5) | +++ (2/5), ++ (1/5), + (1/5) | - | - |
|  | congestion | +++ (5/5) | +++ (2/5), ++ (2/5) | - | - |
|  | swelling | +++ (5/5) | +++ (1/5) ++ (3/5) + (1/5) | - | - |

Different tissues of the piglets (lung, tonsil, brain, kidney and submandibular lymph nodes) were collected and subjected to pathological examination at 14 days post-challenge (dpc), +++, severe; ++, moderate; +, mild; -, none.
